# Creative Music Therapy and Neurodevelopmental Outcomes in Pre-term Infants at 2 Years: A Randomized Controlled Pilot Trial

**DOI:** 10.3389/fped.2021.660393

**Published:** 2021-06-18

**Authors:** Friederike Barbara Haslbeck, Hans Ulrich Bucher, Dirk Bassler, Cornelia Hagmann, Giancarlo Natalucci

**Affiliations:** ^1^Department of Neonatology, Newborn Research Zurich, University Hospital Zurich, Zurich, Switzerland; ^2^Department of Neonatology and Pediatric Intensive Care, University Children's Hospital Zurich, Zurich, Switzerland; ^3^Children's Research Center, University Children's Hospital Zurich, Zurich, Switzerland; ^4^Larsson-Rosenquist Foundation Centre for Neurodevelopment, Growth and Nutrition of the Newborn, University Hospital Zurich, Zurich, Switzerland

**Keywords:** pre-maturity, creative music therapy, randomized controlled trial, outcome, neurodevelopment, Bayley-III, neurological exam

## Abstract

Impaired neurodevelopment is increasingly recognized as a major health issue in children born prematurely. Creative music therapy (CMT) intends to prevent and or reduce neurobehavioral deficits in pre-term infants using musical stimulation and socio-emotional co-regulation. We conducted a randomized, clinical pilot CMT trial to test feasibility and to examine long-term neurodevelopmental outcomes in pre-term infants (NCT02434224: https://clinicaltrials.gov/ct2/show/NCT02434224). Eighty-two pre-term infants were randomized either to CMT or standard care. A specially trained music therapist provided family-integrating CMT via infant-directed singing during hospitalization. Fifty-six infants underwent follow-up at 2 years of corrected age. No significant beneficial nor adverse effects of CMT were identified in routine clinical neurodevelopmental measures (Bayley-III Scales of Infant and Toddler Development and the standardized neurological examination). Longer term follow-up (5 years) and larger future studies are recommended to elucidate possible long-term effects of music in relation to more sensitive outcomes including executive function, detailed language processing and social-emotional development.

## Highlights

- First randomized study to evaluate creative music therapy in pre-term infants.
- Use of standardized, long-term, clinically relevant outcomes for neurodevelopment.
- No significant effect of CMT was identified at 2 years of age.
- Follow-up neurodevelopmental examination at 5 years is recommended.
- Future multi-center investigations with more sensitive outcome measure are needed.

“*R. has developed very well. He does not speak yet (except for mama, tata etc.), but he has been humming lots of songs since May. One can recognize the melody immediately when he starts. All of our children have always liked music, but we have never seen it like him. We always ask ourselves whether early music therapy has influenced it or whether it would have happened otherwise? Sometimes he wakes up at night and can then hum songs for half an hour. Then we have a great concert. Do you have experiences or feedback from parents who report the same thing? We just wonder*… 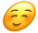
”

Mother's quote of a 1-year-old infant born prematurely in the 24rd week of gestation participating in the present trial.

## Introduction

Impaired neurodevelopment is a major health issue in children born prematurely worldwide. The most frequently observed developmental problems in this high-risk population include cognitive deficits (1, 2), problems with executive function (3, 4) and behavioral issues (5, 6). Developmental problems may lead to academic underachievement and long-life mental health concerns (4, 7). Some children experience severe motor and sensory disabilities including cerebral palsy, hearing loss, and visual impairment (8). Neurodevelopmental follow-up programs for very pre-term infants have become a standard of post-discharge care in many western countries to support early detection and initiate timely supportive therapies.

In addition to many predictors of unfavorable neurodevelopmental outcomes in pre-term infants (e.g., brain lesions, bronchopulmonary dysplasia, infections, retinopathy of prematurity), it is plausible that the stress of the neonatal intensive care unit (NICU) environment and the separation from the mother may negatively influence its development (9–11). Animal and human studies have shown that adequate auditory stimulation as well as socio-emotional caregiving and contact is essential for neurodevelopment (12–15). Toddlers who received active music sessions beginning at 6 months of age show enhanced pre-linguistic communicative, musical, and social development (16). Further evidence indicates that music interventions may enhance neurobehavioral abilities that are often impaired in infants born prematurely (17). Notably music interventions are associated with enhanced self-regulatory abilities in full-term infants (18).

An increasing number and variety of therapies and interventions are emerging. Interventions aim to prevent and reduce neurobehavioral deficits in pre-term infants (19). Creative Music Therapy (CMT) uses musical stimulation and socio-emotional co-regulation to address patient needs. Socio-emotional co-regulation refers to caregiver-guided dyadic process aimed at regulating an infant's emotional distress with musical parameters and therapeutic responsiveness. CMT is an individualized, interactive, resource- and needs-oriented approach with a core tenet that almost every human being responds to music–no matter how premature, ill, or disabled (20). The approach aims to relax and stimulate the infant by involving both the infant and parent(s) in creating meaningful interactions through music. After assessing infant and parental needs, the music therapist hums and sings in lullaby style with entrained responsiveness (21) in parallel to the infant's breathing patterns and in harmony with facial expressions and gesticulations. CMT aims to support infant self-regulation by experienced co-regulation and mutual responsiveness. Because CMT integrates the family, parents are individually in the therapeutic process, i.e., providing music therapy during skin-to-skin contact to foster bi-directional relaxation, bonding and attachment.

Several studies have demonstrated beneficial effects of music therapy on arousal, behavior and infant respiratory rate (22–24). A meta-analysis (25) supports the observed beneficial effects, particularly on infant respiratory rate and maternal anxiety. However, there is little data on the effect of music therapy on short- and long-term neurodevelopment in pre-term infants. To address this gap, we conducted a prospective, randomized, controlled pilot trial examining feasibility and short- and long-term neurodevelopmental outcomes in very pre-term infants (26). The primary outcome of the trial was brain structure and function and assessed by magnetic resonance imaging (MRI) at term equivalent age. The lagged, resting-state MRI analysis revealed infants who have received CMT had more reliable functional networks, higher functional integration, and shorter thalamocortical processing delays in several regions (left prefrontal, supplementary motor, inferior temporal) compared to infants in the control group (27). These promising results suggest potential positive impact on long-term neurodevelopment as these brain regions are associated with discriminating sounds, language, cognition, fine motor coordination and empathy (28, 29). Herein, we report the predefined secondary long-term neurodevelopmental outcomes of the trial participants at 2 years of corrected age (26). We hypothesized that infants receiving CMT would have improved cognitive, language, and motor outcomes compared to the infants who received standard care.

## Materials and Methods

### Study Design

The study is a prospective, single-center, between-subject randomized, controlled pilot trial in preparation for a multi-center trial. The study was conducted in line with the principles of the declaration of Helsinki. The study was reviewed and approved by the Ethics Committee at the University of Zurich, Switzerland (KEK-ZH 2014-0655) and the independent Monitoring Committee of the Swiss Agency for Therapeutic Products (Swissmedic) (Audit No.: CTCQA14). All parents provided written informed consent prior to the initiation of study procedures.

### Population

Eighty-two very pre-term infants were recruited from January 2015 to December 2017 from a level-3 perinatal center (Department of Neonatology, University Hospital Zurich, Switzerland). Infants were eligible if they were born prematurely (gestational age <32 weeks), reached the chronological age ≥ seven days of life and did not require invasive cardiovascular and respiratory support at recruitment. Neonates were excluded if they were diagnosed with a major congenital anomaly/ genetically defined syndrome, congenital malformation adversely affecting life expectancy/neurodevelopment, intraventricular hemorrhage (≥Volpe grade 3) (30), or when the infants were admitted for palliative care. For safety reasons, CMT would pause when infants would require invasive cardiovascular and respiratory support during the study period as recommended in the clinical practice protocol (31).

### Randomization and Masking

Participants were randomly assigned to one of two groups in a 1:1 ratio using a random computer-generated list created prior to study initiation. Allocation was concealed. Blinding of participating families and personnel was not possible due to the nature of the intervention (i.e., integrating parents). Follow-up assessors and data analysts were masked to infant group allocation. Details on the study protocol including blinding for the subsequent long-term follow-up assessment have been previously published (26).

### Procedures

Infants were assigned to receive either standard care according to department practice (including skin-to-skin care), or to receive standard care plus a minimum of 8 CMT sessions as previously recommended for measurable impact (32, 33). Procedures to minimize contamination have been described in the study protocol (26).

Following parental consent, a qualified music therapist (FH) provided CMT for ~20 min at the bedside (incubator or warmer) two to three times per week until hospital discharge. Interventions followed feeding time and were conducted with the infant alone or with the parents providing skin-to-skin contact.

During CMT (infant only), the music therapist started the session by touching the infants, e.g., at the head and feet, to “welcome” the infant and entrain the breathing rhythm (31). After observation, the music therapist would hum smoothly and tailor vocalizations in line with the infant‘s breathing rhythm, facial expression, and gesture. Throughout the session a melody progressively develops according to the infant‘s behavioral state. For example, the therapist would use sedating musical parameters (e.g., calm, repetitive humming) to soothe an agitated infant. Conversely, the therapist would use activating musical parameters (up-rising melodies) to stimulate a limp infant. In all cases, parental musical preferences would be integrated into the improvisation – see clinical CMT protocol for details (31). At the end of the session, the music therapist would smoothly fade out the humming smoothly and gently remove her hands from the infant.

In the presence of the parents, the music therapist uses the same method of humming and singing. During these sessions, a parent holds their infant providing skin-to-skin contact throughout the session. The music therapist places an arched NICU-monochord with 29 strings (Wooden vibro-acoustic string instrument provided by Saitenklang, Bern, Switzerland^©^) at the parents' elbow to transmit relaxing vibrations via bone and air conduction. The music therapist then hums/sings in harmony with the monochord sounds. At the end of the session, the music therapist slowly smoothly fades the music out. Throughout the sessions parents are encouraged and empowered to respond to the infant's cues and needs, as well as to hum and speak “motherese” (34) to facilitate bonding. Prior to hospital discharge, parents receive a consultation providing information and instruction on musical interventions during the first year of life including recommended songs to support musical development for each developmental milestone (27).

### Neonatal Data

Neonatal data was extracted from the prospective national database of the Swiss Neonatal Network. Gestational age, birth weight z-scores, major brain injury, bronchopulmonary dysplasia, retinopathy of prematurity, necrotizing enterocolitis, neonatal sepsis and socioeconomic status were defined as previously published for this cohort (35).

### Neurodevelopment Assessment

At follow-up, parents were invited to bring their child for routine follow-up examinations according to the Swiss post-discharge neurodevelopment surveillance standard for very pre-term infants (36). Experienced developmental pediatricians, who were masked to study group allocation, performed neurodevelopmental assessments (18–24 months of corrected age) at a Swiss Neonatal Network and Follow-up Group Center. The examination involved standardized neurodevelopmental assessments including the Bayley-III Scales of Infant and Toddler Development (Bayley-III) (37) and a structured neurologic examination, somatic growth measurements. Vision and hearing were assessed either by direct examination or via parental report. Cognitive, language, and motor developmental scores were obtained from the Bayley-III. The cognitive composite score includes evaluation of sensorimotor development, object relatedness, memory, exploration, manipulation, concept formation and memory. The language composite score is obtained by summing the receptive and expressive skills scores. The receptive language score includes comprehending and responding to requests as well as discriminating between environmental sounds. The expressive language score evaluates the child's ability to name objects and actions, respond to questions, communicate wants, and use multiword sentences. Similarly, the motor assessment includes both fine motor score (e.g., visual tracking, use of a “pincer” grasp and reaching objects) and a gross motor score (e.g., locomotion, balance, and jumping) (37). Receptive and expressive language scores, and fine and gross motor scores have normative values (standard deviation [SD]) of 10 and composite scores of 100. Higher values indicate better performance. Cerebral palsy was defined and graded according to Rosenbaum et al. (38) and the Gross Motor Function Classification System (GMFCS) (39). Ongoing developmental therapies (e.g., physiotherapy) were recorded according to parental report.

### Outcomes

Prespecified secondary outcomes include Bayley-III cognitive, language and motor development scores as well as cerebral palsy of any grade (present/not present), severe cerebral palsy (GMFS grade 3–5), ongoing therapy, and visual or hearing problems–defined as moderately reduced vision or unilateral blindness with good vision in the contralateral eye and hearing loss (40–90 dB hearing level) corrected with aids, respectively.

*Post-hoc* exploratory outcomes included the composite outcome category “severe neurodevelopmental impairment” which was defined as one of the following: a score below −2SD from the norm (i.e., <70) in the Bayley-III, cerebral palsy with a GMFCS of more than two, visual or hearing problems; and as the continuous absolute values and z-scores of body weight, height, and head circumference. De-identified outcome data were extracted from the Swiss Neonatal record base and entered into the study database for blinded analysis.

### Statistics

The sample size for this pilot trial was set at 60 participants per pilot trial design recommendations (40–42). Since this is the first randomized study to evaluate possible neurodevelopmental effects of CMT in pre-term infants, we employed a pilot design to evaluate outcome measures and assess clinical/recruitment feasibility to determine estimate sample size for a future, appropriately powered trial (26). Independent Student's *t*-test or Mann-Whitney U-test was used to compare baseline characteristics between groups as appropriate. Fisher's exact test was used for to assess interval scaled and nominal variables respectively. Intention-to-treat (ITT) analysis was employed to compare secondary and exploratory outcomes according to treatment allocation. Unadjusted intervention association with outcomes was calculated using independent Student's *t*-test for continuous variables and Fisher's exact test for nominal variables. The assumption that continuous outcome values were normally distributed in both groups was not violated. A per-protocol analysis was conducted by comparing infants who received the treatment originally allocated with a minimum of eight CMT sessions with infants who received standard care according to department practice without CMT (compare 26). Results are reported as mean differences and odds ratios with 95% confidence intervals. Since missing data were related to the severity of prematurity (i.e., not random), no data were imputed. Two-sided tests were performed and findings were considered significant when *p* < 0.05. Statistical analyses were performed by using IBM SPSS, Version 26. The study is reported according to the CONSORT Guidelines (43).

## Results

### Population and Feasibility

Among 293 very pre-term infants, 82 were included in the study (gestational age = 28.0 ± 2.1 weeks). One hundred and twelve infants did not meet all inclusion criteria. As described in our previous paper on study feasibility (27), seventy one parents rejected participation because they were concerned that the MRI exam could be an additional stressful event for their infant and/ or their infant could be assigned to the control group without CMT. In total, 37 were randomly assigned to receive CMT and 45 to standard care (27). Of the 82 randomized infants, eight infants in the standard treatment group were transferred to another hospital. Seven infants from the CMT group did not receive the intervention because they had to be transferred to another center where the CMT intervention was not available. Six infants from the standard care group had to be excluded from the study since the parents withdrew study participation when the infants were randomized to the control group without any music stimulation (compare 27).

All CMT infants received the CMT sessions as planned (median 15; range: 8–30 sessions; total CMT sessions: 446) during hospitalization (median 5 weeks; range: 3–10 weeks). On average, CMT infants received nine CMT sessions during kangaroo-care (range: 4–22; total sessions: 262) and six sessions at the bedside with the infant alone (range 2–18; total sessions: 173). No adverse reactions occurred during and directly after the CMT [compare Haslbeck et al. (27)].

**Figure 1:**
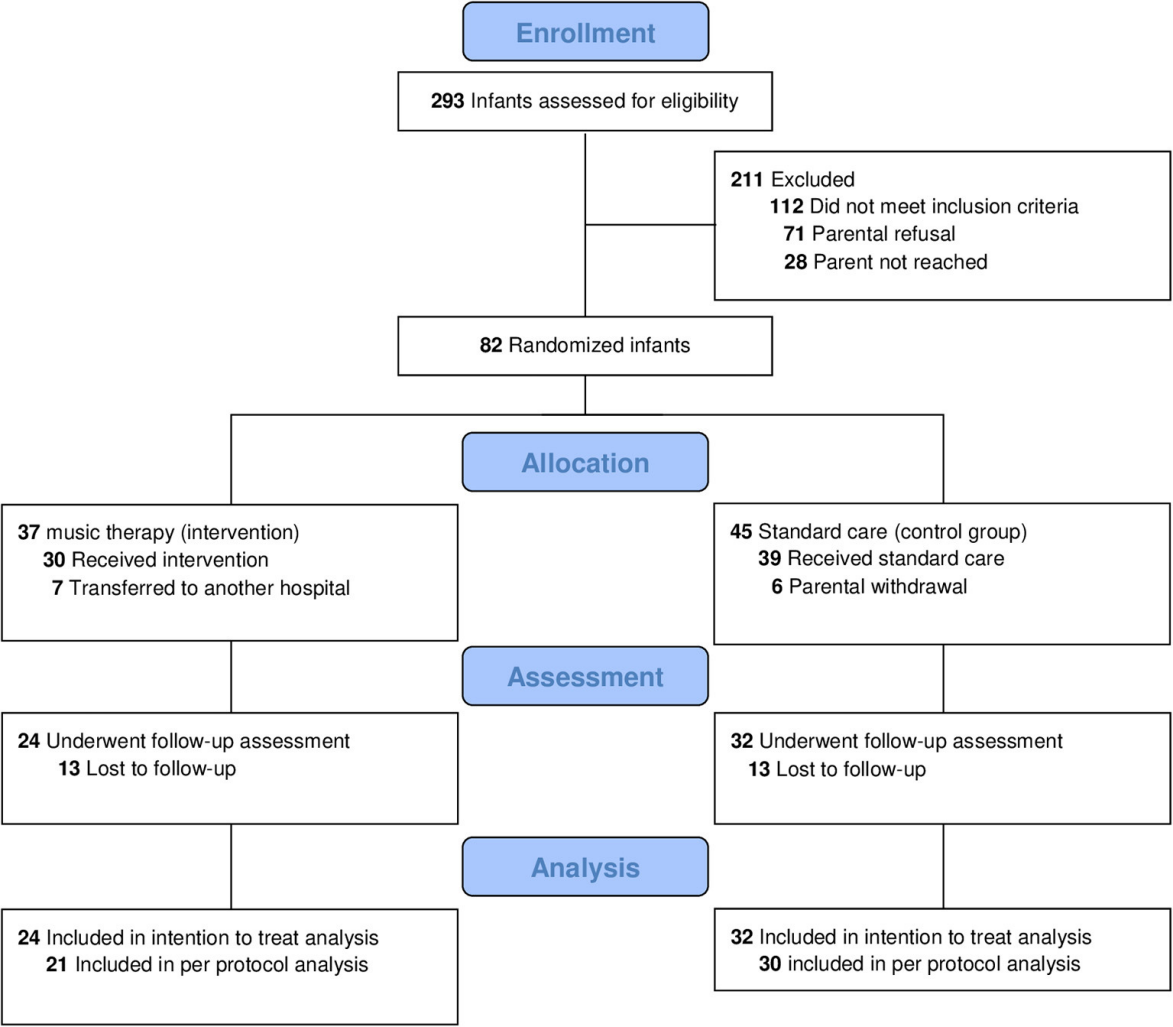
Flow diagram.

Between April 2018 and March 2020 (last assessment), 56/82 (68%) of randomized infants returned for the follow-up visit. Of the 26 drop-outs (*n* = 13 in both the CMT and standard care groups) discontinuation resulted from moving abroad (*n* = 2) or for unknown reasons (*n* = 24) (Figure 1). Groups neither differed in neonatal parameters nor baseline demographic characteristics (Table 1). Compared to infants lost to follow-up, infants assessed at age 2 years had similar baseline characteristics but for lower gestational age (*p* < 0.001), longer supplemental oxygen therapy (*p* = 0.001) and longer hospital stay (*p* < 0.001) (Supplementary Table 1).

**Table 1:** Demographic and neonatal baseline characteristics of infants randomized to music therapy (CMT) or standard care (control group).

	**Music (*n* = 37)**	**Control (*n* = 45)**	***P*-value**
Gestational age at birth (weeks), (mean ± SD)	28.1 ± 1.8	27.8 ± 2.3	0.50
Birth weight (grams), (mean ± SD)	1060 ± 287	1063 ± 358	0.97
Z-score	−0.14 ± 0.73	−0.03 ± 0.86	0.25
Head circumference at birth (cm), mean ± SD	25.8 ± 2.0	25.3 ± 2.6	0.4
Z-score	−0.26 ± 0.60	−0.35 ± 0.69	0.99
Mechanical ventilation (days), mean ± SD	2.6 ± 4.4	3.1 ± 4.0	0.65
Oxygen supplementation (days), mean ± SD	42.0 ± 33.8	35.9 ± 29.5	0.38
Parental socio-economic score (range: 2–12), mean ± SD	5.3 ± 2.4	5.5 ± 2.6	0.69
Sex (female), *n* (%)	13 (35)	21 (47)	0.29
Small for gestational age (i.e., birth weight <10 percentile), *n* (%)	3 (8.1)	3 (6.7)	1.00
Retinopathy of pre-maturity, *n* (%)	0 (0)	3 (6.7)	0.25
Sepsis, *n* (%)	6 (16)	4 (9)	0.34
Bronchopulmonary dysplasia, *n* (%)	7 (8)	8 (9)	0.89
Intraventricular hemorrhage, *n* (%)	4 (5)	7 (8)	0.75
Duration of hospitalization (days), mean ± SD	59.1 ± 27.9	57.4 ± 31.4^*^	0.80

*SD, standard deviation*;

* *n = 44*.

### Neurodevelopmental Outcomes at Corrected Age 2 Years

In the intention-to-treat analysis, no differences were observed between groups in Bayley-III scores, cerebral palsy rate, ongoing developmental therapy, or vision/hearing problems (Table 2). Similarly, exploratory outcomes “neurodevelopmental impairment” rate and somatic growth parameters did not differ at follow-up (Table 2). Findings were confirmed by per protocol analysis (Table 3).

**Table 2:** Intention to treat outcomes at follow-up (2-years) for infants in music (*n* = 24) and standard care (*n* = 32) groups.

**Outcome**	**Music group**	**Control group**	**Odds ratio**	***P*-value**
**Neurodevelopmental outcome, mean** **±** **SD**	**(*****n*** **=** **23)**	**(*****n*** **=** **29)**	**(95% CI)**	
Cognitive composition score	102.4 ± 12.3	102.8 ± 12.6	−0.37 (−7.38–6.69)	0.92
Language composition score	92.4 ± 13.5	91.62 ± 17.1	0.08 (−7.98–9.52)	0.86
Receptive language score	9.4 ± 2.6	8.4 ± 2.8	1.01 (−0.50–2.53)	0.19
Expressive language score	8.0 ± 2.5	8.7 ± 3.2	−0.77 (−2.41–0.87)	0.35
Motor composition score	97.7 ± 12.7	97.6 ± 11.8	0.12 (−6.73–6.97)	0.97
Fine motor score	10.7 ± 2.4	11.0 ± 2.4	−0.26 (−1.59–1.07)	0.70
Gross motor score	8.5 ± 2.8	8.4 ± 1.8	0.05 (−1.26–1.36)	0.94
Cerebral palsy, *n* (%)	1 (4)	1 (3)	0.74 (0.04–12.50)	1.000
GMFCS <2, *n* (%)	22 (96)	28 (100)^*^	0.97 (0.87–1.04)	0.45
Therapy ongoing, *n* (%)	4 (17)	2 (6)	0.33 (0.56–2.00)	0.39
Visual problems, *n* (%)	2 (8)	1 (3)	0.35 (0.03–4.16)	0.39
Hearing problems, *n* (%)	0	0	n/a	n/a
Neurodevelopmental impairment, *n* (%)	8 (33)	10 (31)	0.91 (0.29–2.82)	0.87
**Somatic growth, mean** **±** **SD**	**(*****n*** **=** **23)**	**(*****n*** **=** **30)**	**(95% CI)**	
Weight (kg)	12.3 ± 1.5	12.5 ± 2.3	−0.24 (−1.36–0.88)	0.67
Z-score	0.10 ± 0.90	0.24 ± 1.31	−0.14 (−0.78–0.50)	0.66
Length (cm)	88.4 ± 4.8^**^	87.8 ± 4.7	0.60 (−2.01–3.22)	0.65
Z-score	0.04 ± 0.89	−0.05 ± 1.06^*^	0.09 (−0.48–6.59)	0.75
Head circumference (cm)	51.1 ± 10.3	50.2 ± 9.3	0.90 (−4.42–6.22)	0.74
Z score	−0.16 ± 1.30	−0.42 ± 1.56	0.26 (−0.56–1.09)	0.53

*SD, standard deviation; CI, Confidence interval; GMFCS, Gross Motor Functions Classification System; n/a, not applicable*;

* *n = 28*;

** *n = 24; n = 22*.

**Table 3:** Per protocol outcomes at follow-up (2-years) for infants in music (*n* = 24) and standard care (*n* = 32).

**Outcome**	**Music group**	**Control group**	**Odds ratio**	***P*-value**
**Neurodevelopmental outcome**	**Mean** **±** **SD**	**Mean** **±** **SD**	**(95% CI)**	
Cognitive composition score	102.2 ± 13.2^*^	101.1 ± 10.0^**^	1.14 (−5.68–7.97)	0.74
Language composition score	93.3 ± 14.2^*^	91.4 ± 17.7^**^	1.86 (−7.82–11.53)	0.70
Receptive language score	9.4 ± 2.8^*^	8.4 ± 2.9^**^	1.08 (−0.60–2.76)	0.20
Expressive language score	8.2 ± 2.6^*^	8.7 ± 3.3^**^	−0.47 (−2.27–1.33)	0.60
Motor composition score	97.4 ± 12.7^*^	98.1 ± 11.9^**^	−0.71 (−7.98–6.56)	0.85
Fine motor score	10.9 ± 2.3^*^	11.1 ± 2.3^**^	−0.16 (−1.53–1.21)	0.81
Gross motor score	8.1 ± 2.7^*^	8.5 ± 1.8^**^	−0.35 (−1.71–1.01)	0.61
Cerebral palsy, *n* (%)	1 (5)	1 (3)	0.69 (0.04–11.68)	1.00
GMFCS <2, *n* (%)	19 (95)^*^	26 (100)^‡^	0.95 (0.86–1.05)	0.44
Ongoing therapy, *n* (%)	4 (19)	2 (7)	0.30 (0.05–1.84)	0.21
Visual problems, *n* (%)	2 (9)	1 (3)	0.33 (0.03–3.87)	0.56
Neurodevelopmental impairment, *n* (%)	7 (33)	9 (30)	0.86 (0.26–2.84)	0.80
**Somatic growth**				
Weight (kg)	12.2 ± 1.5^*^	12.4 ± 2.16^†^	−0.22 (−1.36–0.92)	0.70
Z-score	0.02 ± 0.93^*^	0.21 ± 1.19^†^	−0.19 (−0.83–0.46)	0.56
Length (cm)	88.3 ± 5.01^***^	87.5 ± 4.34^†^	0.728 (−1.91–3.47)	0.56
Z-score	−0.06 ± 0.89^‡^	0.00 ± 1.06^**^	−0.057 (−0.65–0.539)	0.85
Head circumference (cm)	51.2 ± 11.1	50.2 ± 9.6^°^	1.020 (−4.88–6.92)	0.73
Z Score	−0.31 ± 1.37^*^	−0.47 ± 1.59^†^	0.158 (−0.73–1.04)	0.72

*SD, standard deviation; CI, Confidence interval; GMFCS, Gross Motor Functions Classification System*;

* *n = 20*;

** *n = 27*;

*** *n = 24*;

‡ *n = 26*;

† *n = 28*;

° *n = 29*.

## Discussion

We report prespecified secondary neurodevelopmental outcomes of a randomized controlled pilot trial investigating the effect of CMT on brain development at term equivalent age. No treatment effects of CMT were noted on cognitive, language and motor outcomes in 2-year-old pre-term infants. These findings are in contrast with the trial primary outcome analysis of the trial. Primary outcome analysis at term equivalent age indicated evidence of beneficial CMT effects on functional brain activity and connectivity in networks underlying higher-order cognitive, socio-emotional, and motor functions (27). In line with the present study, a recent report noted no group differences in Bayley-III findings between infants exposed to recorded music in the NICU compared to controls (44). Similar to the primary outcomes of our study, investigators identified enhanced brain network development at term equivalent age in the intervention group (45).

We expected that CMT may positively influence receptive auditory and language skills in the intervention group, particularly in comprehending and responding to requests as well as in discriminating environmental sound. This assumption was based on a prior report of fMRI findings of improved superior temporal gyrus functional connectivity in infants receiving CMT–linked with language comprehension and processing of social cognition (46). Additionally, we have previously reported improved centrality in the left and right hippocampus with CMT (27)–associated with social-emotional processing, comprehension and response (47). Observed correlations are aligned with the assumption that music and language processing activate similar networks (48). Researchers have posited the capacity to receive, comprehend, and discriminate sound stimuli depends on the hearer‘s prior hearing experiences (49, 50).

One explanation for the lack of observed CMT effect observed in this pilot trial may be that CMT has no effect on the long-term development of the infant. Another possible explanation may be that the standardized neurodevelopment battery may not be sensitive enough to detect changes that are observed in the fMRI analysis. Lejeune and colleagues additionally used the Laboratory Temperament Assessment Battery an additional outcome measurement alongside the Bayley-III. However, no differences were observed in pre-term born infants randomized to music exposure vs. controls (no music) at 12 and 24 months. The effect of CMT on neurocognitive development may be better explored with test batteries examining executive functioning. Sensitive endpoints evaluating composite emotional processing and regulation, executive functioning and detailed language processing may be more suitable for assessing the possible impact of music on neurodevelopment. This is relevant as music has been specifically linked to non-musical faculties including socio-emotional competencies, intelligence, and language processing (51). Our group and others have reported increased functional connectivity in brain regions is associated with neonatal music exposure in pre-term infants (27, 45). Future research should explore hot (i.e., driven by emotion) and cold (i.e., driven by logic and critical analysis) executive functioning (52), working memory (53), and selective attention (54).

Alternative explanations for present findings include the fact that the standardized neurodevelopmental exam during early infancy does not assess the wide range of cognitive functions. Indeed, such cognitive functions, working memory, self-regulation and language skills appear then differentiate during development from early infancy to school age such as. Some have argued that the Bayley-III test underestimates developmental delay in very pre-term infants assessed at 2 years of age thereby reducing statistical power of randomized controlled trials (55). While the Bayley-III may be appropriate for providing evidence on the safety of early intervention, researchers have questioned the predictive validity of the Bayley-III for examining efficacy at 2 years of age -when severely disabled infants have been excluded – as was the case in the present study (41, 56, 57). Therefore, we plan long-term neurodevelopment assessment at school age as neurodevelopment can be tested in a more sensitive and specific way (57, 58).

Another possible explanation for the lack of long-term effect of CMT on neurodevelopment may be that the intervention was exclusively provided during the NICU stay. A post-discharge follow-up music therapy program comprising music group sessions and home visits by a qualified music therapist could fill this gap and bridge specialized NICU care and ambulatory pediatric care while supporting parent-to-parent (peer) in group sessions–as recommended by several authors (59, 60). Several review articles note that early intervention bridging hospital with follow-up interventions are associated with most beneficial outcomes (19).

In general, the question remains if our improved MRI results at term equivalent age (TEA) in the CMT infants may indicate improved long-term outcomes in this population. Indeed, abnormalities detected in MRI exams at TEA are claimed to be associated with adverse long-term outcomes in pre-term infants (61). Woodward et al. (8) have demonstrated significant links between cerebral white-matter and gray-matter abnormalities in MRI results at TAE and adverse neurodevelopmental outcomes, e.g., severe psychomotor and cognitive delay, at 2 years of age in very pre-term infants. However, the authors emphasize that impairments at TAE must not result in adverse long-term outcomes since further environmental, therapeutic, and educational factors may influence the infant‘s neurodevelopment. Besides, Van't Hooft et al. (61) conclude in their meta-analysis that MRI results at TEA may not predict other neurocognitive and behavioral long-term outcomes in pre-term infants. Further research may be needed to shed more light on strong associations between MRI results at TEA and long-term outcomes among pre-term infants in general and in particular concerning music interventions (8, 44, 61).

In terms of study feasibility, our findings show that CMT is safe and readily implemented. Overall study participation and compliance were moderate (27). It is worthwhile to note that the single-center study setting did not provide optimal conditions for conducting a randomized treatment intervention. Study participants were hospitalized in the same NICU and some parents of in the standard care group indirectly experienced the nature of the CMT. For this reason, some parents decided to withdraw participation and some parents may have indirectly acquired some CMT input. To mitigate this potential risk in a future trial, cluster randomization may be considered as an alternative approach to reduce the risk of cross-contamination between groups and improve compliance rates.

The drop-out rate at follow-up exam was high (32%) despite the fact that the Swiss neonatal network assures invitations to all eligible very pre-term infants with continuously updated address lists (36). The high attrition rate is in line with national (35) and international (36) attendance rates that vary from 50 to 100% (62). Like in the study by Schlapbach et al. (35), a number of infants with milder prematurity symptoms missed the follow-up exam resulting in a slight underestimation of these participants. To improve parental follow-up compliance, e.g., for the planned multi-center study, (63) recommend that neonatologists should earlier inform parents about the value of the follow-up exams, to invite the parents via phone and letter, and to invite twins/ triplets to the same appointment. When parents do not attend the exam the responsible physician should call the parents and declare the value of the exam again (36).

There are some study limitations that decrease the generalizability of our findings. First, this a pilot study had a small sample size thereby reducing the power of secondary outcome analyses. Second, although drop-outs were evenly distributed between groups, differences in attrition rates at follow-up may have introduced bias in evaluating outcomes. Nevertheless, feasibility and pilot testing results provide key information informing a planned multi-center study.

A relative strength of this study is that CMT is non-invasive, low-cost, and family-integrating approach and we used standardized routine long-term clinically relevant measures of neurodevelopment to evaluate the intervention. Future trials could investigate parental psychological adjustment and bonding as preliminary evidence suggests that CMT contributes to parental empowerment and activates “communicative musicality” (64) thereby benefiting parent-infant-attachment (21, 65, 66). Interventions that support parent-infant attachment in early life may enhance infant socioemotional development, mental health and future relationships (67, 68). This is particularly salient for pre-term infants who may have suboptimal attachment circumstances due to NICU stays (9). Moreover, successful parenting may be one of the most valuable resource for the child health and psychosocial development (19).

## Conclusion

This randomized pilot trial examining the effect of CMT on neurodevelopment in pre-term infants at 2 years shows no evidence of treatment effect. These observations are in contrast with results of the primary outcome analysis of brain MRI imaging data at term equivalent age. We plan further follow-up examination at 5 years, when the neurodevelopment is more differentiated. Future studies with larger samples with sensitive outcome measures of executive functioning, language processing, social-emotional development, quality of life and parent-infant-bonding may uncover possible mid- and long-term effects of CMT on infants and their parents.

## Data Availability Statement

The original contributions presented in the study are included in the article/Supplementary Material, further inquiries can be directed to the corresponding author.

## Ethics Statement

The studies involving human participants were reviewed and approved by Ethics committee Zurich. Written informed consent to participate in this study was provided by the participants' legal guardian/next of kin.

## Author Contributions

FH: conceptualization, data curation, statistical analysis, funding acquisition, investigation, methodology, project administration, resources, software, visualization, and writing–original draft. DB and HB: conceptualization, supervision, validation, and writing–review. CH: writing–review. GN: conceptualization, main supervision, visualization, writing–main review, and editing. All authors contributed to the article and approved the submitted version.

## Conflict of Interest

The authors declare that the research was conducted in the absence of any commercial or financial relationships that could be construed as a potential conflict of interest.

## Abbreviations

Bayley-III: Bayley Scales of Infant and Toddler Development, third edition
CCS: Cognitive composite score
CMT: Creative music therapy
GMFCS: Gross motor function classification system
ITT: Intention-to-treat analysis
NICU: Neonatal intensive care unit
MCS: Motor composite score
MRI: Magnetic resonance imaging
PP: Per-Protocol analysis
TEA: Term equivalent age.
