# Supplementary material for: Creative Music Therapy and Neurodevelopmental Outcomes in Pre-term Infants at 2 Years: A Randomized Controlled Pilot Trial
Source: Front Pediatr. 2021 Jun 18;9:660393. doi: 10.3389/fped.2021.660393 (PMC8249730; doi:10.3389/fped.2021.660393)
Supplement: Supplementary file 1 [file Table_1.DOCX]

|  | **Follow-Up (n = 56)** | **No Follow-Up (*n* = 26)** | ***P*-value** |
| --- | --- | --- | --- |
| Gestational age at birth (weeks), mean ± SD | 27.4 ± 2.1 | 29.2 ± 1.4 | <.001 |
| Birthweight (grams), mean ± SD | 979 ± 320 | 1241 ± 264 | <.001 |
| Z-score | -0.48 ± 1.50 | -0.326 ± 0.682 | .32 |
| Head circumference at birth (cm), mean ± SD | 24.9 ± 2.5 | 26.8 ± 1.5 | <.001 |
| Z-score | -0.33 ± 0.68 | -0.264 ± 0.586 | 0.53 |
| Mechanical ventilation (days), mean ± SD | 3.4 ± 4.7 | 1.7 ± 2.4 | .089 |
| Oxygen supplementation (days), mean ± SD | 42.0 ± 33.8 | 35.9 ± 29.5 | .001 |
| Total parental socio-economic scores (range 2-12), mean ± SD | 5.5 ± 2.8 | 5.2 ± 2.3 | .63 |
| Sex (female), n (%) | 25 (30) | 9 (11) | .39 |
| Small for gestational age (<10 percentile), n (%) | 6 (7) | 0 | .17 |
| Retinopathy of prematurity, n (%) | 3 (5) | 0 | .55 |
| Sepsis, n (%) | 8 (14) | 2 (8) | .49 |
| Bronchopulmonary dysplasia, n (%) | 11 (13) | 4 (4) | .7^7^ |
| Intraventricular hemorrhages, n (%) | 9 (11) | 2 (2) | .4^9^ |
| Duration of hospitalization (days), mean ± SD, | 67.9 ± 28.6^*^ | 37.8 ± 20.2 | <.001 |

**Supplemental table 1: Demographic and clinical parameter comparison of infants assessed with or without 2 years neurodevelopmental exam (FU2)**

SD, standard deviation;^*^ n = 55
